# CD44v, S1PR1, HER3, MET and cancer‐associated amino acid transporters are promising targets for the pancreatic cancers characterized using mAb

**DOI:** 10.1002/2211-5463.13963

**Published:** 2025-01-05

**Authors:** Takashi Nakano, Kouki Okita, Shogo Okazaki, Soshi Yoshimoto, Sachiko Masuko, Hideki Yagi, Kazunori Kato, Yoshihisa Tomioka, Kenichi Imai, Yoichi Hamada, Kazue Masuko, Kayoko Shimada‐Takaura, Noriaki Nagai, Hideyuki Saya, Tomio Arai, Toshiyuki Ishiwata, Takashi Masuko

**Affiliations:** ^1^ Cell Biology Laboratory, School of Pharmacy Kindai University Higashiosaka‐shi Japan; ^2^ Faculty of Health and Sports Sciences Toyo University Kita‐ku Japan; ^3^ Advanced Design for Pharmaceuticals, School of Pharmacy Kindai University Higashiosaka‐shi Japan; ^4^ Department of Microbiology, Division of Immunology and Pathobiology, School of Dentistry Nihon University Chiyoda‐ku Japan; ^5^ PIERAS Co., Ltd Osaka‐shi Japan; ^6^ Department of Pharmaceuticals, Faculty of Pharmacy International University of Health and Welfare Otawara‐shi Japan; ^7^ Oncology Pharmacy Practice and Science, Graduate School of Pharmaceutical Sciences Tohoku University Sendai‐shi Japan; ^8^ Hamada Clinic Higashiosaka‐shi Japan; ^9^ Natural Drug Resources, Faculty of Pharmacy Kindai University Higashiosaka‐shi Japan; ^10^ Oncology Innovation Center Fujita Health University Toyoake‐shi Japan; ^11^ Division of Aging and Carcinogenesis, Research Team for Geriatric Pathology Tokyo Metropolitan Institute for Geriatrics and Gerontology Itabashi‐ku Japan

**Keywords:** amino acid transporter, CD44v, HER3, MET, pancreatic cancer, S1PR1

## Abstract

Effective therapies have yet to be established for pancreatic ductal adenocarcinomas (PDAC) even though it is the most aggressive cancer. In the present study, PDAC was analyzed using novel rat mAbs against membrane proteins in conjunction with flow cytometry and immunohistochemistry. Human epidermal growth receptor (HER)1–4, mesenchymal to epithelial transition factor (MET), sphingosine‐1‐phospahate receptor 1 (S1PR1), l‐type amino acid transporter 1 (LAT1), system x^−^_c_ transporter (xCT), alanine‐serine‐cysteine transporter (ASCT2), cationic amino acid transporter 1 (CAT1) and variant CD44 (CD44v) were expressed at high frequencies in both *in vitro* and *in vivo* PDAC. Internalization of membrane proteins by mAbs and growth inhibition by toxin‐linked mAbs were demonstrated in many PDAC cell lines, and mAbs against S1PR1, ASCT2, HER3 and CD44v inhibited the growth of xenografted MIA PaCa‐2 PDAC cells. Furthermore, CD44v‐high PDAC showed high mRNA expression of HER1–3, MET and CD44v, and was correlated with poor prognosis. Taken together, our results suggest that CD44v, S1PR1, HER3, MET and the above‐mentioned cancer‐associated amino acid transporters might be promising targets for the diagnosis and treatment of PDAC.

## Graphical abstract

Novel mAbs could detect cancer‐associated membrane proteins on various type of pancreatic ductal adenocarcinomas (PDAC) by flow cytometry with viable PDAC cells and by immunohistochemistry with PDAC tissues. Results of protein expression were substantiated by mRNA expression by The Cancer Genome Atlas. PDAC will be handled by targeting mAb‐defined single or combinations of multiple molecules such as variant CD44 (CD44v), sphingosine‐1‐phospahate receptor 1 (S1PR1), human epidermal growth receptor (HER)1–4, mesenchymal to epithelial transition factor (MET), l‐type amino acid transporter 1 (LAT1), system x^−^_c_ transporter (xCT), alanine‐serine‐cysteine transporter (ASCT2) and cationic amino acid transporter 1 (CAT1).

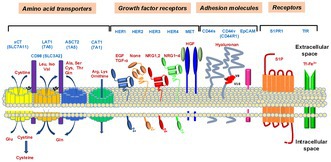

**Abbreviations**
ADCC: antibody‐dependent cellular cytotoxicity
ASCT2: alanine‐serine‐cysteine transporter
CAT1: cationic amino acid transporter 1
CD44v: variant CD44
EGFR: epidermal growth factor receptor
EpCAM: epithelial cell adhesion molecule
FCM: flow cytometry
GFP: green fluorescent protein
HER: human epidermal growth factor receptor
IHC: immunohistochemistry
LAT1: L‐type amino acid transporter
MET: mesenchymal to epithelial transition factor
MFI: mean fluorescence intensity
NEC: neuroendocrine carcinomas
PBMC: peripheral blood mononuclear cell
PBS: phosphate‐buffered saline
PE: phycoerythrin
PCA: principal component analysis
PDAC: pancreatic ductal adenocarcinoma
RTK: receptor tyrosine kinase
S1PR1: sphingosine‐1‐phosphate receptor 1
TCGA: The Cancer Genome Atlas
TfR: transferrin receptor
xCT: system x^−^_c_ transporter

Global estimates toward 2070 predict increases in colorectal, breast and lung cancers, as well as prostatic cancer [1], and effective molecular targeted therapies and immunotherapies are actively being developed. Targeted therapies against human epidermal growth factor receptor/human epidermal growth factor receptor (EGFR/HER) family receptor tyrosine kinases (RTKs) [2] have been successfully applied to various malignancies, such as head and neck, lung, breast and colorectal cancers [3, 4, 5, 6, 7]. HER family RTKs (HER1/EGFR/*erb*B‐1, HER2/*erb*B‐2/*neu*, HER3/*erb*B‐3 and HER4/*erb*B‐4) can form homodimers or heterodimers, transmit various intracellular events, such as the mitogen‐activated protein kinase and phosphoinositide 3‐kinase‐Akt pathways [8, 9] and are recognized as attractive targets for cancer therapy [10, 11].

By contrast to the major cancers described above, effective therapies have yet to be established for pancreatic ductal adenocarcinoma (PDAC), even though it is the most aggressive cancer with poor patient survival and is one of the leading causes of cancer death worldwide [12]. Although multimodal therapies using surgery, radiotherapy and classical chemotherapy are generally the main treatment approach for PDAC, novel targeted therapies need to be actively pursued. Molecular targeted therapies against HER1 (low‐molecular‐weight RTK inhibitors such as erlotinib) with chemotherapeutics, including gemcitabine, paclitaxel, irinotecan, oxaliplatin, 5‐fluorouracil and immune checkpoint inhibitors, such as pembrolizumab and nivolumab, are administered to patients with PDAC [13]; however, their efficacy is very limited.

We have continuously reported the successful production, characterization and application of mAbs [14, 15] that recognize the extracellular domains of cancer‐associated membrane proteins, including the HER family (HER1 [14], HER2 [16, 17, 18], HER3 [19, 20, 21, 22] and HER4 [23]) and mesenchymal to epithelial transition factor (c‐MET or MET) [24] RTKs, transferrin receptor (TfR) [25, 26], amino acid transporters {l‐type amino acid transporter 1 (LAT1) [27, 28, 29, 30], system x^−^_c_ transporter (xCT) [31, 32, 33, 34], alanine‐serine‐cysteine transporter (ASCT2) [35, 36] and cationic amino acid transporter 1 (CAT1) [37]}, sphingosine‐1‐phosphate receptor 1 (S1PR1) [38], epithelial cell adhesion molecule [epithelial cell adhesion molecule (EpCAM) [14, 15], and variant hyaluronate receptors [variant CD44 (CD44v) [39, 40, 41, 42].

In the present study, we characterized *in vitro* and *in vivo* PDAC using mAbs against membrane proteins including above‐mentioned growth factor receptors, transporters and adhesion molecules expressed on living PDAC cell lines and PDAC tissue sections.

## Materials and methods

### Animals

Female F344 rats and male KSN nude mice were obtained from the Shimizu Animal Farm (Kyoto, Japan). All animals were maintained under specific pathogen‐free conditions and were housed individually in plastic cages under a standard light/dark photocycle at a constant temperature of 23 ± 1 °C. Animal care and experiments in the present study were approved by the Committee for the Care and Use of Laboratory Animals, School of Pharmacy, Kindai University (KAPS‐23‐004). At the end of the animal maintenance, isoflurane euthanasia of rats and mice was performed using a vaporizer with continued exposure to isoflurane for at least 15 min after respiratory arrest was acceptable, as reviewed by IACUC (https://animal.research.uiowa.edu/iacuc‐polycy‐confirmation‐euthanasia).

### Cell culture

Eleven human PADC cell lines (PANC‐1, PK‐1, PK‐8, PK‐45P, PK‐59, T3M‐4, MIA PaCa‐2, KP‐4, BxPC‐3, AsPC‐1 and Capan‐1), three immortalized pancreatic epithelial cell lines (CRL‐4023, CRL‐4037/wild‐type KRAS and CRL‐4038/G12D‐mutated KRAS), and P3X63Ag8.653 and P3U1 mouse myelomas were obtained from ATCC (Manassas, VA, USA) or Japanese Collection of Research Bioresources Cell Bank (Osaka, Japan). RH7777 rat hepatoma cells were kindly donated by K. Chiba (Mitsubishi Tanabe Pharma, Osaka, Japan). Peripheral blood mononuclear cells (PBMC) were prepared from healthy individuals by using PluriMate (Pluriselect‐USA, Inc., El Cajon, CA, USA) and cultured with or without recombinant human interleukin‐2 (500 JRU·mL^−1^; Shionogi Pharma Co., Ltd, Osaka, Japan). Treatment of human blood samples was approved by the Research Ethics Committee, School of Pharmacy, Kindai University (approval no. 22‐221) with the written informed consent of the participants, and was conducted in accordance with the Declaration of Helsinki. These cells and HEK293F (Invitrogen, Carlsbad, CA, USA) were cultured in RD medium [43] containing equal volumes of RPMI‐1640 and Dulbecco's modified Eagle's medium (Nacalai Tesque Inc., Kyoto, Japan) with heat‐inactivated 7% fetal bovine serum (Invitrogen) in a humidified incubator (5% CO_2_) at 37 °C. Aseptic processing was strictly controlled using MediAir air purifiers (Pieras Co., Ltd., Osaka‐shi, Japan).

### Primary mAbs

Besides IM7 (Invitrogen), which recognizes pan‐CD44 (CD44p), all rat mAbs were produced by Masuko *et al*. [14, 15, 24, 38]. F344 rats were immunized three to six times with RH7777 rat hepatoma cells expressing human target proteins fused to green fluorescent protein (GFP). Spleen cells from immunized rats were fused with P3X63Ag8.653 or P3U1 mouse myelomas, and selected and cloned hybridomas secreting mAbs against target proteins were established. These mAbs were screened for reactivity with RH7777 and HEK293F transfectants expressing GFP‐fused target proteins in a GFP intensity‐dependent manner [14, 15, 27, 38]. We used specific rat mAbs against HER1 (Ab83‐3), HER2 (Ab6‐5), HER3 [19, 20, 21, 22] (Ab1, Ab3, Ab4 and Ab6), HER4 (P6‐1), MET (Ab57), l‐type amino acid transporter 1/LAT1 (Ab1‐27 and Ab1‐31), system x^−^_c_ transporter/xCT (Ab2‐31), alanine‐serine‐cysteine transporter/ASCT2 (Ab38), cationic amino acid transporter 1/CAT1 (CA2), epithelial cell adhesion molecule/EpCAM (1D12), variant‐type CD44/CD44v8 (RV14), CD44v9 (RV3 and RV10), CD98 (HR35), sphingosine‐1‐phosphate receptor 1 (S1PR1 and YQS), and transferrin receptor (TfR and Ab135). We also used mouse mAb against CD98 (HBJ127) [44, 45, 46, 47, 48, 49], HER1 (m1B3) [50], HER2 (SER4) [16, 17, 18] and CD44v6 (VFF‐18; Abcam, Cambridge, UK) in the expression analysis. All mAbs were used at final concentrations of 1–80 μg·mL^−1^.

### Flow cytometry (FCM)

Cells (2.5 × 10^5^ to 1.0 × 10^6^) in 50‐μL Dulbecco's phosphate‐buffered saline (PBS) containing 1% BSA (Nacalai) were mixed with primary mAb (50 μL) diluted to 20 μg·mL^−1^ in RD medium (final mAb concentration: 10 μg·mL^−1^) and incubated on ice for 1 h. Cells were incubated with 50 μL of secondary polyclonal antibodies (pAb, 1 : 300) at 4 °C for 30 min. Phycoerythrin (PE)‐labeled donkey anti‐rat IgG (H + L) and PE‐ or Alexa488‐labeled anti‐mouse IgG (H + L) species‐specific donkey pAb (#712‐116‐153, #715‐116‐150 and #715‐547‐003) purchased from Jackson ImmunoResearch (West Grove, PA, USA) were used as secondary pAb. Stained cells were suspended in 0.1% BSA‐PBS, and filtered through a nylon mesh (#352235; BD Falcon, Franklin Lakes, NJ, USA). FCM was performed using LSRFortessa™ X‐20 (BD, Sunnyvale, CA, USA) and analyzed using flowjo, version 10.10) (BD, Tokyo Japan). Relative mean fluorescence intensity (rMFI: MFI with mAb/MFI without mAb) and subtracted MFI (ΔMFI) were calculated from the values of MFI.

### Internalization activity of mAbs against PDAC cell lines

Pancreatic ductal adenocarcinoma cells (1 × 10^5^) were suspended in 100 μL of RD medium with or without rat mAbs (10 μg·mL^−1^) and incubated at 37 °C or 4 °C for 1 h. After washing of with PBS, cells were mixed with 1 : 300 diluted PE‐conjugated anti‐rat IgG (H + L) (Jackson ImmunoResearch) in 1% BSA‐PBS on ice for 30 min. After washing, cell‐surface fluorescence was analyzed by FCM. Internalization (%) was calculated using the formula: [1 − (ΔMFI at 37 °C/ΔMFI at 4 °C) × 100].

### Growth inhibition by rat mAbs against PDAC cell lines with Saporin‐conjugated secondary antibodies

Rat mAb solution (4 μg·mL^−1^, 25 μL), a PDAC cell suspension (3 × 10^4^ cells·mL^−1^, 50 μL) and Saporin‐conjugated goat anti‐rat IgG pAb (Rat‐ZAP Secondary Conjugates; Advanced Targetting Systems, Carlsbad, CA, USA) (4 μg·mL^−1^, 25 μL) were added to each well of 96‐well plates (BD Falcon). This mixture (1.5 × 10^3^ cells, 1 μg·mL^−1^ ZAP, with or without 1 μg·mL^−1^ rat mAb) in 100 μL of 7% fetal bovine serum‐RD medium was incubated in a humidified incubator (5% CO_2_) at 37 °C for 72–96 h. WST‐8‐based Cell Count Reagent SF (Nacalai; 1 : 10 diluted in 7% fetal bovine serum‐RD medium) was added (50 μL per well) and absorbance (450 nm) was measured with a RAINBOW THERMO microplate reader (TECAN Japan, Osaka, Japan). Growth inhibition (%) was quantified using the formula: (1 − OD_450_ with rat mAb/OD_450_ with control rat IgG) × 100.

### Immunohistochemistry (IHC) of human pancreatic tissue sections

Pancreatic cancer with pancreas tissue arrays (PA501 and T141c; US Biomax, Inc., Rockville, MD, USA) were used. Following deparaffinization by xylene and rehydration with ethanol and water, tissue sections were untreated or subjected to antigen‐retrieval (pH 9.0, 98–100 °C, 20 min or proteinase K or 0.2 mg·mL^−1^, 20 °C, 5 min). After three washes with PBS, sections were incubated with 3% H_2_O_2_ in methanol at room temperature for 10 min to quench the effects of endogenous peroxidase activity. After three washes with PBS, sections were incubated with Block Ace (DS Pharma Biomedical Co., Ltd, Osaka, Japan) at room temperature for 2 h. They were then incubated with rat mAbs (20–80 μg·mL^−1^) diluted in 1% BSA‐PBS at 4 °C overnight. After two washes with PBS, the sections were incubated with ImmPress HRP Goat Anti‐Rat IgG Polymer (#MP‐7404 or #MP‐7452; Vector Laboratories, Burlingame, CA, USA) diluted 1 : 200 in 1% BSA‐PBS at room temperature for 30 min. After three washes with PBS, sections were incubated with 0.05% 3,3′‐diaminobenzidine (Dojin Chemicals, Kumamoto, Japan) and 0.01% H_2_O_2_ in 0.1 m Tris–HCl (pH 7.4) and then counterstained with hematoxylin. Sections were dehydrated with ethanol, cleared in xylene and mounted in Permount (Thermo Fisher Scientific, Waltham, MA, USA). IHC scores for the expression of mAb‐stained molecules were assessed by two skillful pathologists (Drs Toshiyuki Ishiwata and Tomio Arai, Tokyo Metropolitan Institute for Geriatrics and Gerontology). IHC gave a score of 0–3, indicating the staining intensities in tissue samples. IHC scores in normal (adjacent to or distant from PDAC) and cancer tissues were estimated as 0 (negative), 1 (weak or borderline), 2 (intermediate) or 3 (strong).

### *In vivo* PDAC tumor therapy using a xenograft model

Nude mice aged 6–8 weeks were randomly separated into five groups. MIA PaCa‐2 cells (5 × 10^6^ cells/mouse) in 0.2 mL of PBS were subcutaneously injected and visible tumors were confirmed in all mice. At this point (day 0), control or focused rat mAbs (100 μg of IgG in 500 μL of PBS) were i.p. injected, followed by an additional injection on day 7. Tumor volumes were measured every 2 days with digital calipers and were quantified using the formula: volume (mm^3^) = [length (mm)] × [width (mm)]^2^ × 0.5.

### The Cancer Genome Atlas (TCGA) and statistical analysis

All TCGA‐Pancreatic Adenocarcinoma (PAAD) data used in this study were downloaded from UCSC Xena (https://xenabrowser.net) [51]. Log_2_‐transformed RPKM and RSEM values were used for CD44 variant exon expression and mRNA expression analysis, respectively. The exons used for CD44 exons are CD44s6: chr11:35236399–35236461: + (plus strand), CD44v6: chr11:35226059–35226187: +, CD44v8: chr11:35229652–35229753: +, CD44v9: chr11:35231512–35231601: +, and CD44v10: chr11:35232793–35232996: +. In a Kaplan–Meier survival analysis of cancer patients with the log rank test, the relationship between mRNA expression and survival was analyzed using prism, version 10.1.10 (GraphPad Software Inc., San Diego, CA, USA).

### Principal component analysis (PCA) and clustering analysis

Principal component analysis and visualization were performed using R (https://www.r‐project.org). PCA was conducted using prcomp function. Two‐dimensional (2D) and three‐dimensional (3D) plots were depicted by biplot or plot3d function in rgl library. Two‐way hierarchical clustering analysis was performed using heatmapper (http://www.heatmapper.ca) [52]. Default settings were used for the distance metric and linkage method, and the results were displayed as a heatmap.

### Statistical analysis

All statistical analyses were performed using Excel (Microsoft Corp., Redmond, WA, USA) and prism (GraphPad Software Inc.). Comparisons between two groups of parametric and non‐parametric data were conducted using Student's *t‐*test and the Mann–Whitney *U*‐test, respectively. One‐way analysis of variance followed by Dunnett's post‐hoc test was used to determine significance for comparisons among multiple groups. Differences in frequency of TNM stages between PDAC clusters were analyzed using the chi‐squared test. *P* < 0.05 was considered statistically significant. Data are presented as the mean ± SEM. In the correlation analysis, data were analyzed using Pearson's correlation coefficient.

## Results

### Comprehensive membrane protein analysis of human PDAC cell lines

**Fig. 1:**
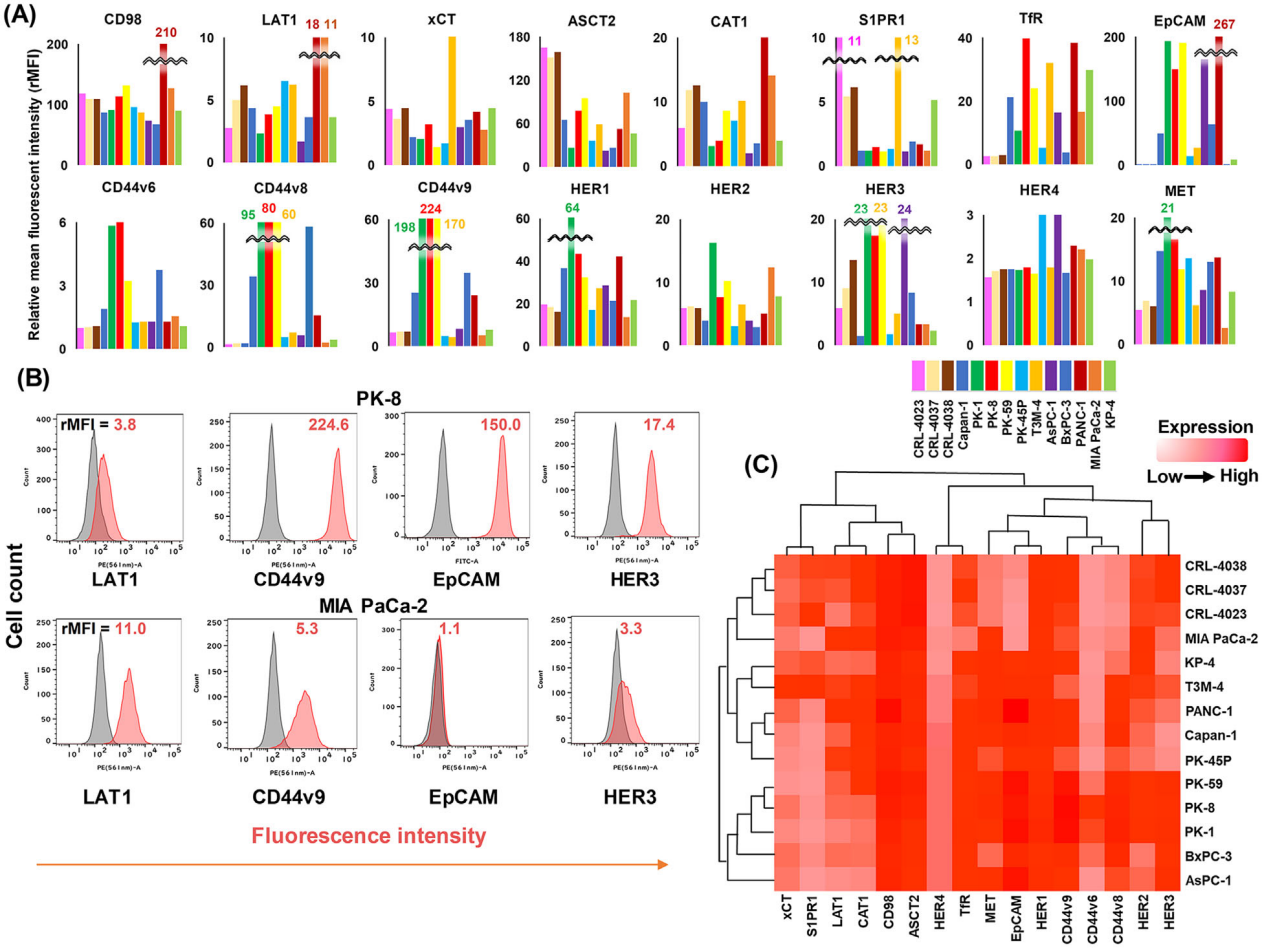
Comprehensive membrane protein analysis of human PDAC cell lines. (A) The expression levels of 16 membrane proteins in 14 pancreatic cell lines were analyzed by FCM, digitized by the values of rMFI and displayed using colored bar graphs. (B) Typical FCM histograms showing the reactivities of anti‐LAT1, anti‐CD44v9, anti‐EpCAM and anti‐HER3 rat mAbs against PK‐8 and MIA PaCa‐2. (C) Heatmap clustering of 14 pancreatic cell lines (columns) and 16 cell membrane proteins (rows). Expression levels were digitized by rMFI from high (red) to low (white).

The expression levels of 16 membrane proteins in 11 living PDAC and three non‐cancerous (CRL) cells are shown by rMFI in FCM (Fig. 1A). Besides ASCT2 and S1PR1, the expression of 14 proteins was higher in some PDAC than in CRL cell lines. Representative FCM histograms with anti‐LAT1, CD44v9, EpCAM and HER3 mAbs against PK‐8 and MIA PaCa‐2 are shown (Fig. 1B). Protein expression levels in various pancreatic cell lines are shown as a clustered heatmap (Fig. 1C). Membrane proteins were grouped into three categories: xCT, S1PR1, LAT1, CAT1, CD98 and ASCT2 (left) belonging to transporters, HER4 and TfR (middle), and MET, EpCAM, HER1, CD44v, HER2 and HER3 (right) belonging to adhesion molecules or growth factor receptors. Pancreatic cell lines were grouped into three categories by this hierarchical clustering: CRL‐4038, ‐4037, ‐4023 and MIA PaCa‐2 (upper), KP‐4, T3M‐4, PANC‐1, Capan‐1 and PK‐45P (middle), and PK‐59, PK‐8, PK‐1, BxPC‐3 and AsPC‐1 (lower).

### Reactivity of mAbs with human PBMC

**Fig. 2:**
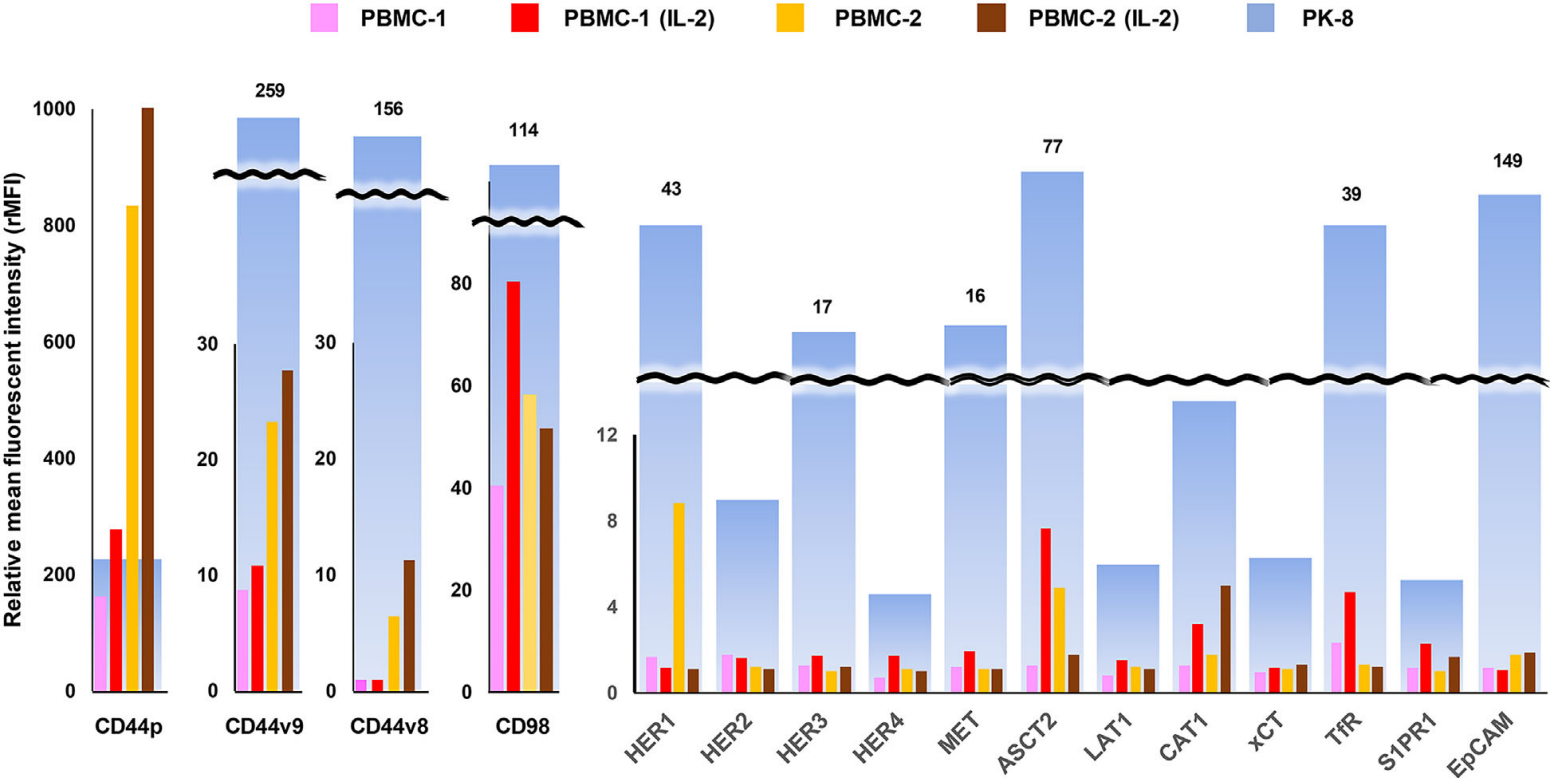
Reactivity of mAbs with human PBMC. The reactivities of mAbs against 16 membrane proteins with human PBMC were analyzed by FCM, and mAb binding to PBMC was shown by rMFI. Resting and activated PBMC were obtained from two healthy adults. rMFI in PDAC (PK‐8) cells are shown for comparison (light blue bar graphs).

To verify the cancer selectivity of mAb, we examined the reactivity of mAbs with freshly prepared human PBMC using FCM (Fig. 2). In comparison with the stronger reactivity of anti‐CD44p mAbs in untreated (resting) and interleukin‐2‐stimulated (activated) human PBMC‐2 than in PK‐8, mAbs against CD44v, CD98, HER family proteins, MET, amino acid transporters (ASCT2, LAT1, CAT1 and xCT), TfR, S1PR1 and EpCAM were more highly reactive in PK‐8 (light blue column) than in resting or activated PBMC from two healthy adults.

### Effects of mAbs against membrane proteins on the internalization activity in PDAC cell lines

**Fig. 3:**
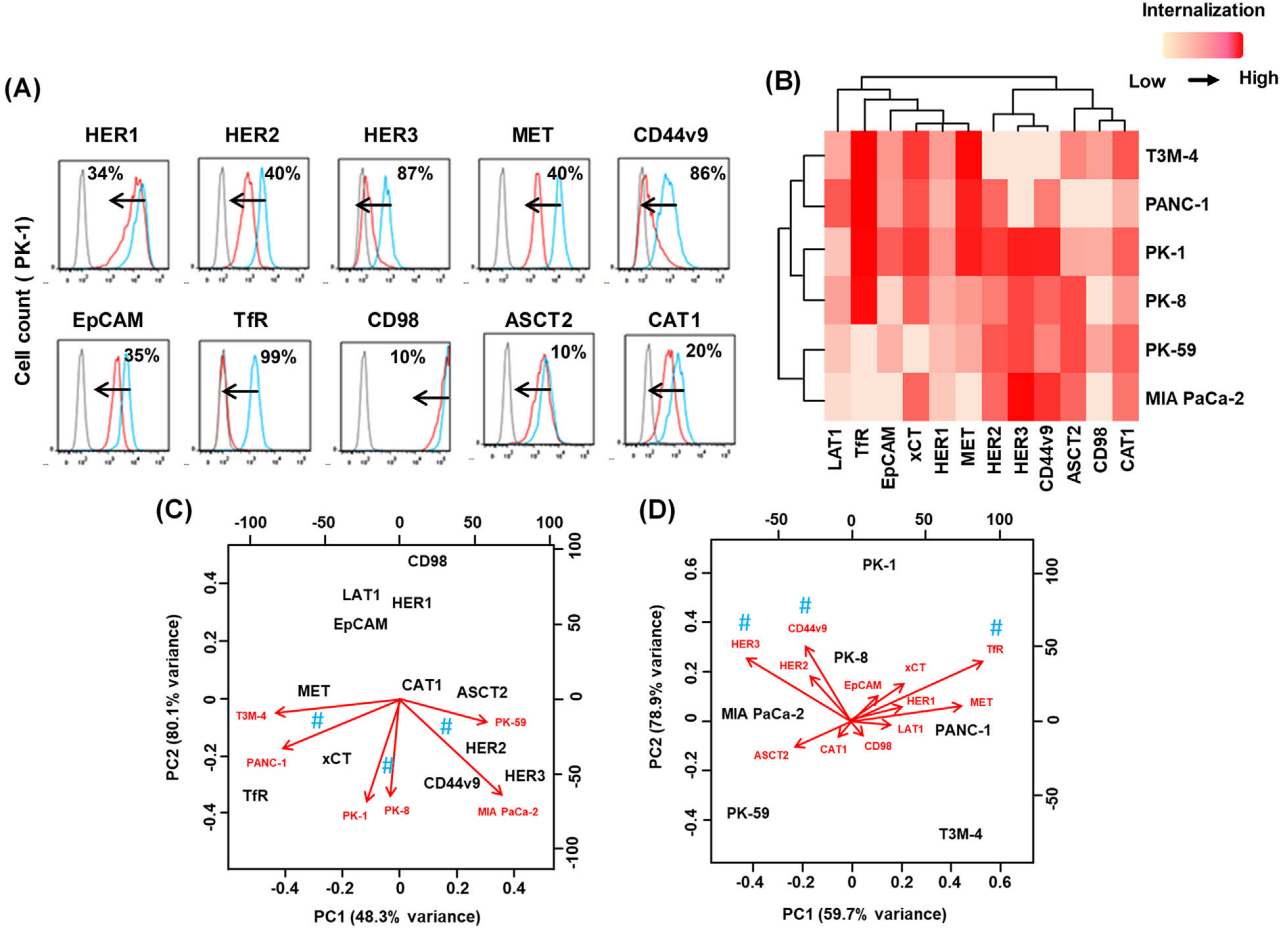
Effects of mAbs against membrane proteins on the internalization activity in PDAC cell lines. Regarding the quantitative analysis of internalization by FCM, the internalization (%) in six PDAC cell lines by 12 mAbs was calculated using the formula [1 − (ΔMFI at 37 °C/ΔMFI at 4 °C) × 100]. (A) Typical FCM histograms (blue lines at 4 °C and red lines at 37 °C) showing the internalization activities of 10 mAbs against PK‐1. (B) Heatmap clustering of 12 human membrane proteins (columns) and six PDAC cell lines (rows) was prepared from mAb‐mediated internalization (%). (C, D) PCA of 12 membrane proteins and six PDAC cell lines was performed with mAb‐mediated internalization (%).

The internalization activity of mAbs against membrane proteins was analyzed by FCM with six PDAC cell lines. These cells were evaluated for mAb‐mediated internalization against 12 membrane proteins (Fig. 3). According to mAb, internalization activity was observed to varying degrees (10–99%) (Fig. 3A) in PK‐1 cells. Heatmap and PCA analyses between the internalization activity of mAbs and PDAC cell types confirmed T3M‐4/PANC‐1, PK‐1/PK‐8 and PK‐59/MIA PaCa‐2 cell clusters (Fig. 3B and C), and the internalization activity of mAbs against TfR, CD44v9 and HER3 was higher than that of mAbs against the other proteins (Fig. 3D).

### Growth inhibitory activity of rat mAbs against PDAC cell lines with Saporin‐conjugated secondary antibodies

**Fig. 4:**
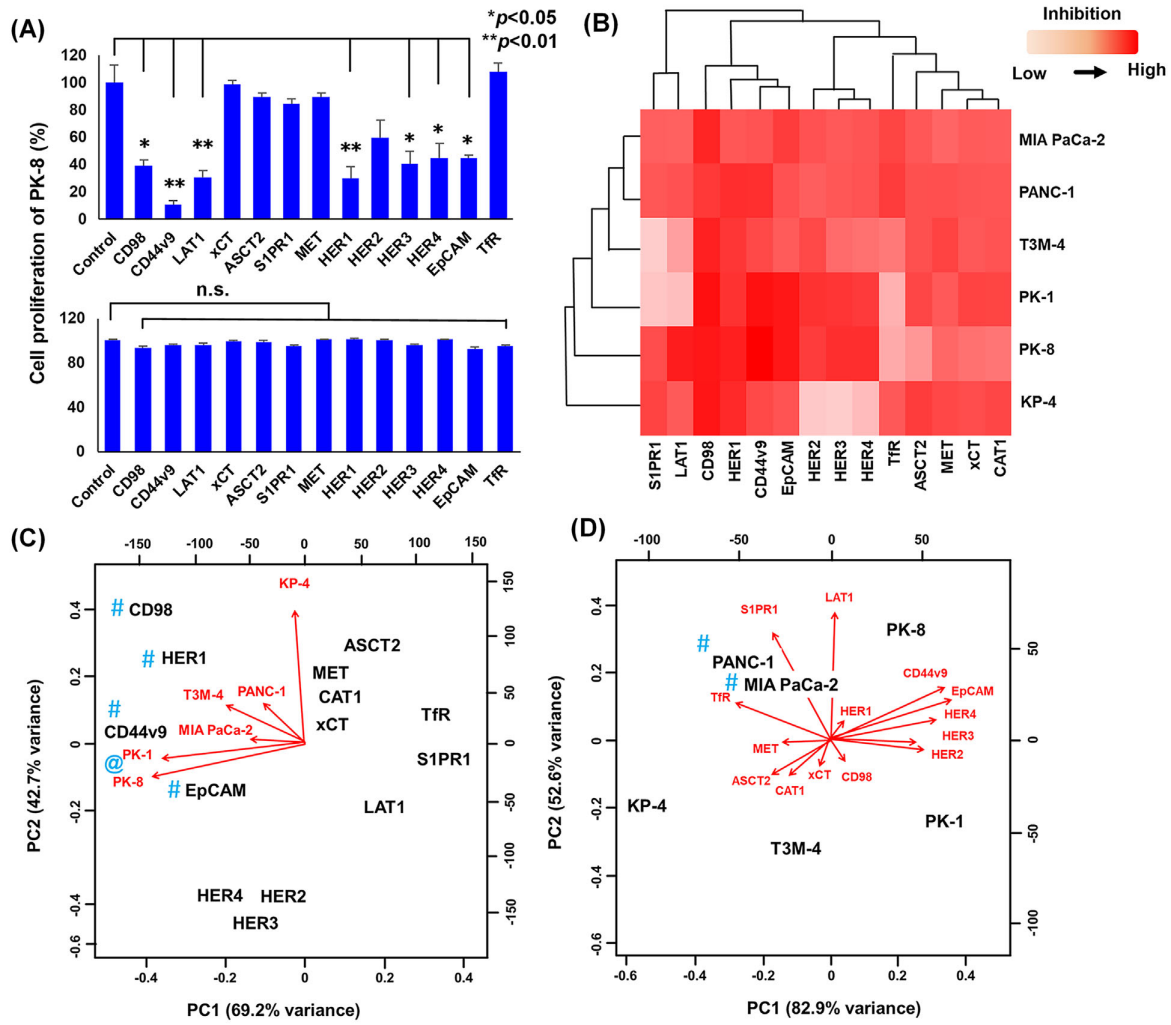
Growth inhibitory activity of rat mAbs against PDAC cell lines with Saporin‐conjugated secondary antibodies. Rat mAbs (1 μg·mL^−1^) were tested for cytotoxic activity with Saporin‐conjugated secondary antibodies (1 μg·mL^−1^) against six representative PDAC cell lines, and the growth inhibition (%) of PDAC was evaluated by the WST‐8 assay. (A) Effects of various rat mAb on PK‐8 cells with (upper) or without (lower) Saporin‐conjugated anti‐rat IgG are shown as bar graphs. Vertical bars show the SEM (*n* = 5) and data were analyzed statistically by Dunnett's test. The final mAb concentration (15 μg·mL^−1^) without Saporin‐conjugated anti‐rat IgG was used (lower part). (B) Heatmap clustering of 14 human membrane proteins (columns) and six PDAC cell lines (rows) prepared from the values of growth inhibition (%). (C, D) PCA analyses of internalization activities of mAbs against 14 membrane proteins with six PDAC cell lines.

Rat mAbs were tested for growth inhibitory activity with Saporin‐conjugated secondary antibodies against six PDAC cell lines (Fig. 4). Growth inhibition (%) by mAbs was quantified by comparison with control growth without mAbs. Rat mAbs against CD98, CD44v9, LAT1, HER1, HER3, HER4 and EpCAM displayed significant Saporin‐mediated growth inhibitory activity in PK‐8 (Fig. 4A, upper) and similar results were observed in PK‐1 via the heatmap (Fig. 4B) and PCA (Fig. 4C) analyses; however, cell growth inhibition was not noted with these mAbs alone (Fig. 4A, lower). Antibodies against CD98, HER1, CD44v9 and EpCAM exerted strong growth inhibitory effects against all six PDAC cell lines (Fig. 4B and C), and all 14 mAbs were widely effective in PANC‐1 and MIA PaCa‐2 cells (Fig. 4B and D).

### Suppressed proliferation of PDAC tumor xenografts by mAbs

**Fig. 5:**
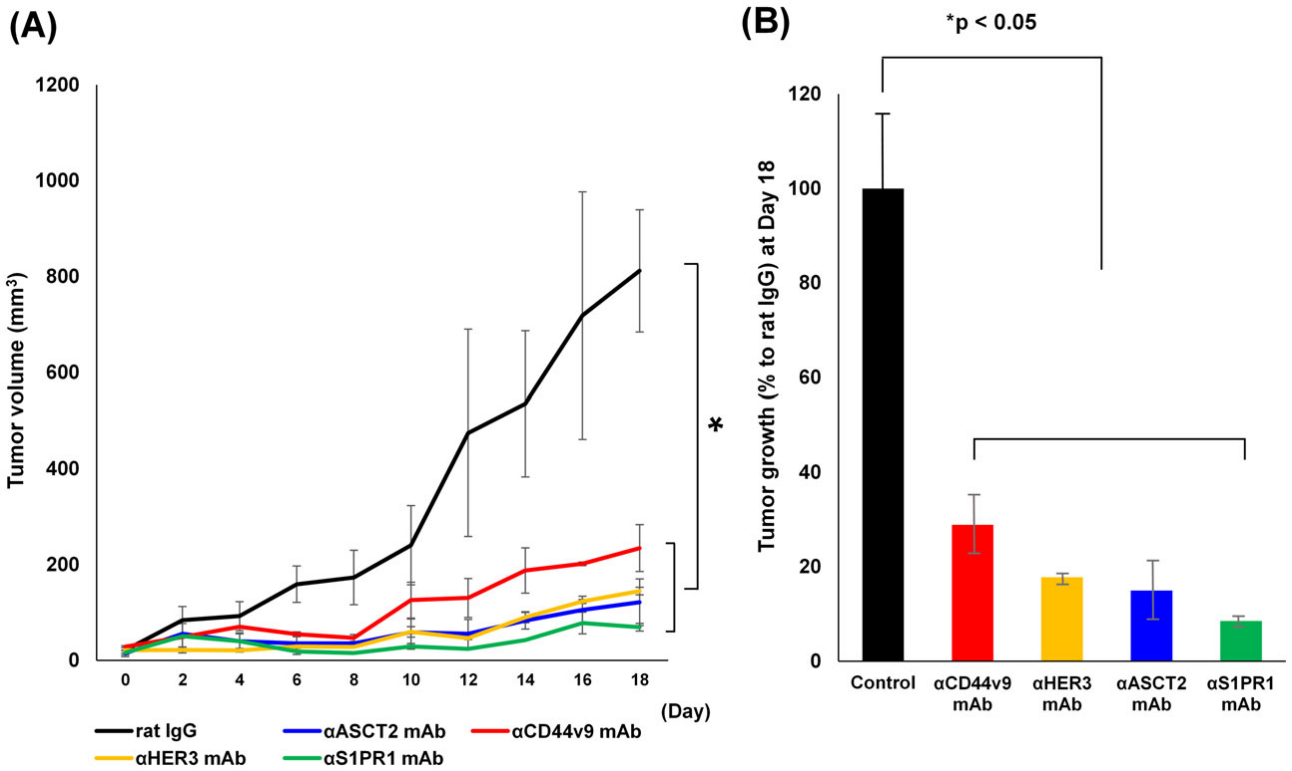
Suppressed proliferation of PDAC tumor xenografts by mAbs. Effects of rat mAbs on the *in vivo* tumor growth of MIA PaCa‐2 cells. Cells (5 × 10^6^ cells) were subcutaneously injected. (A) After visible tumors were confirmed (day 0), mAb (100 μg per mouse) was i.p. injected on days 1 and 7. Tumor volumes were measured every 2 days using digital calipers. (B) Tumor volumes on day 18 are shown by a bar graph (% to control rat IG) and *P*‐values were calculated by Dunnett's test. The results are expressed as the mean ± SEM (*n* = 4).

We assessed the anti‐tumor effects of rat mAbs recognizing CD44v9, HER3, ASCT2 or S1PR1 in the MIA PaCa‐2 PDAC‐xenografted mouse model (Fig. 5). Tumor growth inhibition (%) by the tested mAbs was quantified by the comparison with the growth with control rat mAbs. The anti‐tumor effects of all four mAbs (isotype: γ2a heavy chain, κ light chain) on the *in vivo* growth of MIA PaCa‐2 PDAC cells were verified (*P* < 0.05), and anti‐S1PR1 mAb exerted the strongest anti‐tumor effects.

### Comprehensive membrane protein analysis of human PDAC tissues

**Fig. 6:**
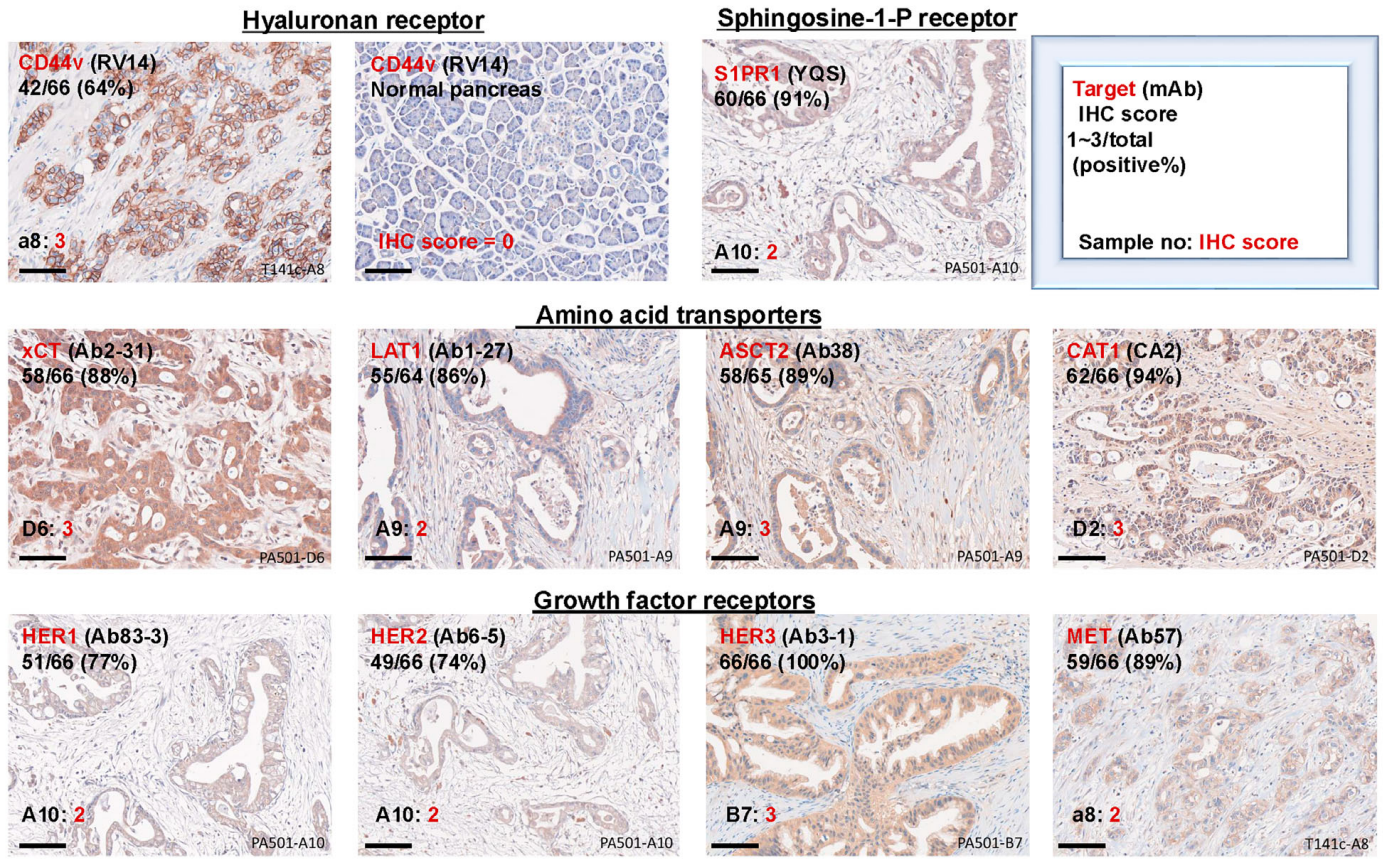
Typical immunostaining of human PDAC tissues by rat mAbs against 10 human membrane proteins. Immuno‐peroxidase staining of human PDAC tissues with 10 selected rat mAbs is shown (Scale bars = 100 μm). The target proteins (mAb designation), positive ratio (%), sample name and IHC scores (1–3: positive) are shown in the upper right corner box, in addition to staining images of PDAC. Negative staining (IHC score 0) by anti‐CD44v8 mAb with normal pancreas tissue is also shown.

**Fig. 7:**
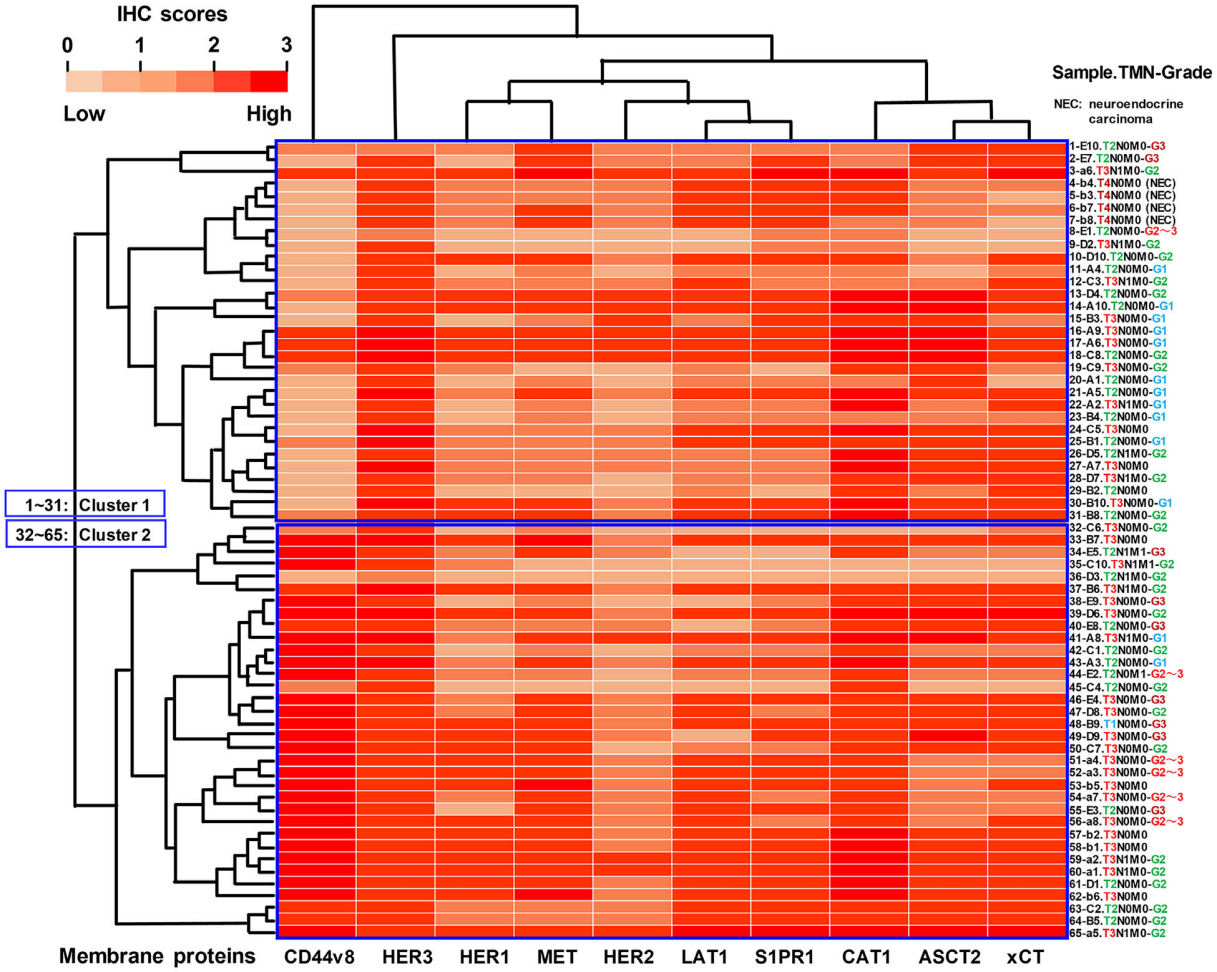
Comprehensive membrane protein analysis of human PDAC tissues with a heatmap analysis. A heatmap of the two‐way hierarchical clustering of 10 human membrane proteins (columns) and 65 PDAC tissue samples (rows) was prepared. The expression levels of membrane proteins were quantified by IHC scores obtained by the reactivity of mAbs.

**Fig. 8:**
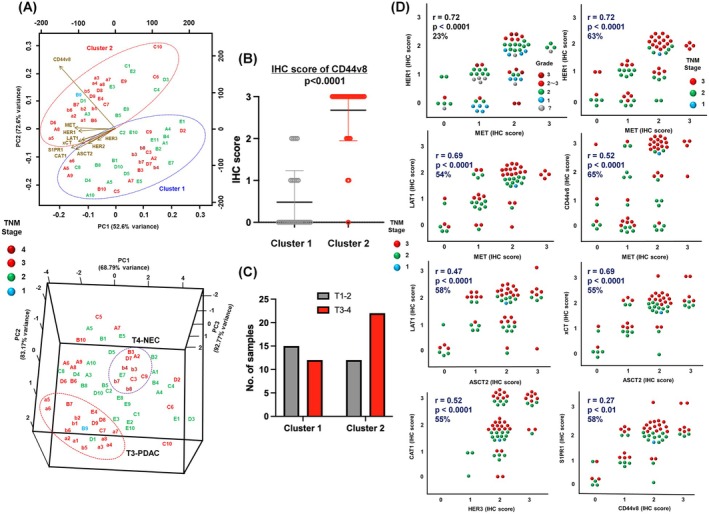
PCA and correlation analyses of PDAC tissues. (A) The expression of membrane proteins in 65 PDAC tissues analyzed by 2D (upper)‐ or 3D (lower)‐PCA. (B) CD44v8 IHC scores between cluster 1 and cluster 2 were compared. Individual *P*‐values were calculated by the Mann–Whitney *U*‐test. Results are expressed as the mean ± SEM. (C) Percentage of T1–T2 and T3–T4 samples in cluster 1 and cluster 2. Individual *P*‐values were calculated using the chi‐squared test, based on the number of T1–T2 and T3–T4 samples within each cluster. (D) Correlation analysis of combinations of several membrane proteins defined with IHC scores by mAbs. The pathological grade (upper left only) and TNM staging of each sample are depicted by colored circles. Pearson's correlation coefficient (*r*), *P*‐values, and IHC scores 2 and 3 in both molecules (%) were calculated.

Based on preliminary experiments with three mouse and 20 rat mAbs recognizing 15 cell surface proteins (HER1–4, MET, LAT1, xCT, CAT1, ASCT2, CD98, S1PR1, TfR, EpCAM, CD44v8 and CD44v9), rat mAbs against 10 membrane proteins (HER1, HER2, HER3, MET, LAT1, xCT, CAT1, ASCT2, S1PR1 and CD44v8) were selected because they definitely and frequently immunostained PDAC cells in pancreatic tissue sections. Representative staining images, positive ratios and IHC scores are shown (Fig. 6) and high positive ratios (64–100%) in PDAC were observed using these 10 rat mAbs. IHC scores in pancreatic tissue samples were expressed as a clustered heatmap (Fig. 7), and membrane proteins aligned into CD44v8, a left‐sided cluster of growth factor receptors (HER3, HER1, MET and HER2) and a right‐sided cluster of transporters (LAT1, S1PR1, CAT1, ASCT2 and xCT). PDAC tissues were clearly categorized to cluster 1 (no. 1–31) and cluster 2 (no. 2–65), mainly by CD44v8 expression status. Cluster 1 included the T4 stage of neuroendocrine carcinomas (NEC), which are CD44v8‐negative and HER3/LAT1/S1PR1‐high, whereas cluster 2 mainly correlated with the T3 stage and G2–G3 grades. Because the removal of NEC samples had little effect on the molecular alignment in a clustered heatmap, we analyzed both PDAC and NEC as human pancreatic cancers in Fig. 7; however, NEC data are removed in Fig. 8D. PCA analyses (Fig. 8A) also highlighted these clusters (upper, 2D‐PCA) and T3‐rich PDAC and T4‐NEC groups (lower, 3D‐PCA). A higher CD448v IHC score (upper) and higher TNM stage in cluster 2 are shown in Fig. 8B,C. CD44v8 IHC scores were significantly higher in cluster 2 compared to cluster 1 (*P* < 0.0001) (Fig. 8B). Although the difference in TNM stage was not statistically significant, cluster 2 exhibited a tendency toward a higher stage (*P* = 0.1288), suggesting a potential association (Fig. 8C). A positive correlation was noted in the expression between various membrane proteins (Fig. 8D). For example, the HER1‐MET double‐high group was rich in TNM stage 2–3 (63%, *r* = 0.72: high positive correlation, *P* < 0.0001) patients, but not in those with pathological grade 2–3 or 3 (23%). Combination of LAT1‐MET, CD44v8‐MET, xCT‐ASCT2 or CAT1‐HER3 showed moderate positive correlations (TNM stage 2–3: 55–65%, *r* = 0.52–0.69, *P* < 0.0001), whereas LAT1‐ASCT2 (*r* = 0.47, *P* < 0.0001) and S1PR1‐CD44v8 (*r* = 0.27, *P* < 0.01) showed low or negligible correlation, respectively, for the relevant proteins.

### TCGA analysis

**Fig. 9:**
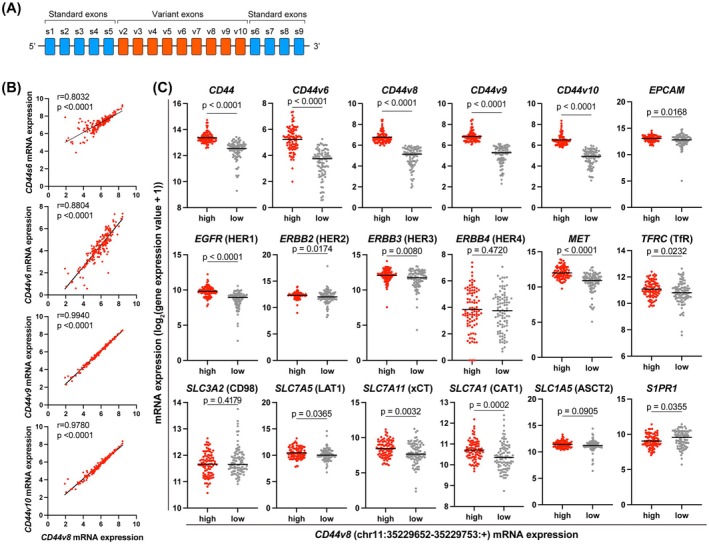
TCGA analysis of the correlation of mRNA expression between CD44v and various membrane proteins in PDAC samples. (A) A schematic diagram of exons comprising CD44. (B) Correlation of mRNA expression levels between CD44 variant exon 8 and other exons (standard exon 6, variant exons 6, 9 and 10) in the TCGA‐PAAD datasets. *r*, Pearson correlation coefficient. (C) Beeswarm plots of the mRNA expression of various membrane proteins in the CD44v8‐high and CD44v8‐low expression groups in the TCGA‐PAAD. The vertical axis ‘Gene expression value’ shows RPKM (i.e. reads per kilobase million) for CD44 variant isoforms v6, v8, v9 and v10, as well as normalized counts for other genes. Because some genes did not follow a normal distribution, *P*‐values were calculated using the Mann–Whitney *U*‐test.

**Fig. 10:**
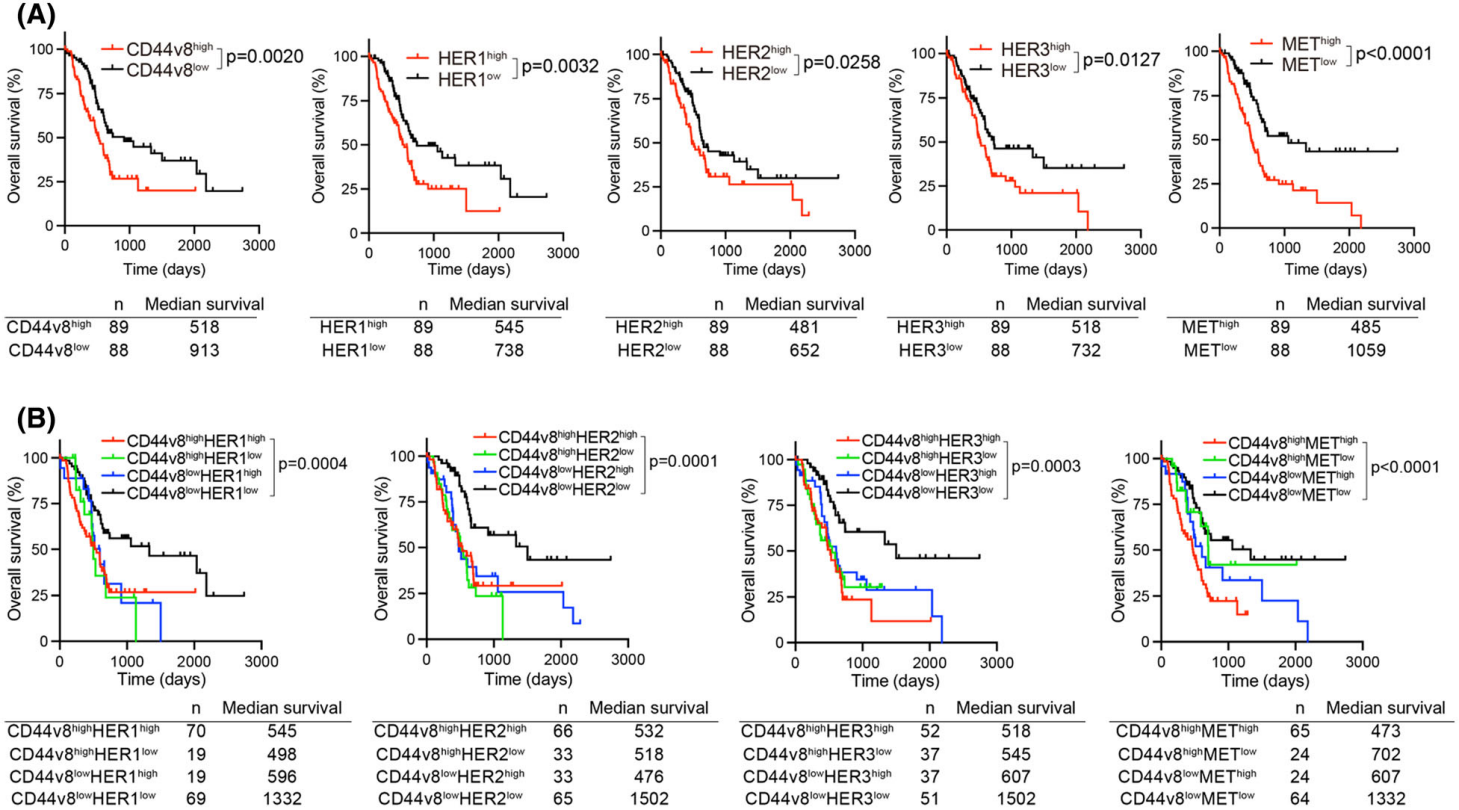
TCGA analysis of PDAC prognosis with the expression of mRNA coding membranes proteins. (A) Kaplan–Meier plots of overall survival in the TCGA‐PAAD, with patients stratified into high and low expression groups for each indicated gene using median expression as a cut‐off. Individual *P*‐values were calculated by log‐rank tests comparing the two groups. (B) Kaplan–Meier plots of overall survival in TCGA‐PAAD, with patients stratified into four groups (high/high, high/low, low/high and low/low) for each indicated gene pair using median expression as a cut‐off. Individual *P*‐values were calculated by log‐rank tests comparing the high/high and low/low groups for each gene pair.

In addition to mAb‐based analyses of *in vitro* and *in vivo* PDAC, we examined the expression of mRNA encoding various membrane proteins (HER1, HER2, HER3, HER4, MET, EpCAM, CD44, CD98, LAT1, xCT, ASCT2, CAT1, S1PR1 and TfR) in PDAC using the TCGA‐PAAD dataset [53]. PDAC proteins were clearly separated into two clusters by CD44v8 expression in the IHC analysis (Figs 7 and 8); therefore, we compared the mRNA expression of CD44 species (Fig. 9A). Our analysis revealed that all exons demonstrated high correlation coefficients with statistically significant differences. Notably, CD44v8, v9 and v10 showed very strong correlations. Although CD44s6 and v6 also exhibited a high correlation with CD44v8, their correlation coefficients were lower compared to those of CD44v9 and v10 (Fig. 9B). Among the various membrane proteins, the higher mRNA expression of *CD44v8* was well correlated (*P* < 0.05) with the higher expression of *EpCAM*, *EGFR* (HER1), *ERBB2* (HER2), *ERBB3* (HER3), *MET*, *TFRC* (*TfR*), *SLC7A5* (*LAT1*), *SLC7A11* (xCT) and *SLC1A5* (CAT1) (Fig. 9C). A negative correlation between *CD44v8* versus *S1PR1* and no correlation of *CD44v8* versus *ERBB4* and *CD98* with respect to mRNA expression were observed. The TCGA‐PAAD dataset revealed the significant involvement of *CD44v8*, *EGFR*, *ERBB2*, *ERBB3* or *MET* in the worse prognoses of PDAC patients (Fig. 10). Furthermore, patients with the double‐high expression of *CD44v8* with *EGFR*, *ERBB2*, *ERBB3* or *MET* were more likely to have poor prognoses.

## Discussion

Because PDAC is the most aggressive cancer with poor patient survival, novel targeted therapies need to be actively pursued. We have recently used a ^64^Cu‐labeled mAb‐targeting system [54] and showed that mAbs against HER1 and TfR exerted high binding and therapeutic effects in PDAC cell lines.

Among the 11 PDAC cell lines examined in the present study, PK‐1, PK‐8, PK‐45P, PK‐59, T3M‐4 and Capan‐1 have been classified as the epithelial type and PANC‐1, KP‐4 and MIA PaCa‐2 as the (quasi‐) mesenchymal type based on an analysis of molecules such as E‐cadherin and vimentin and from electron microscopic images of PDAC cells grown under 2D and 3D culture conditions [55].

**Fig. 11:**
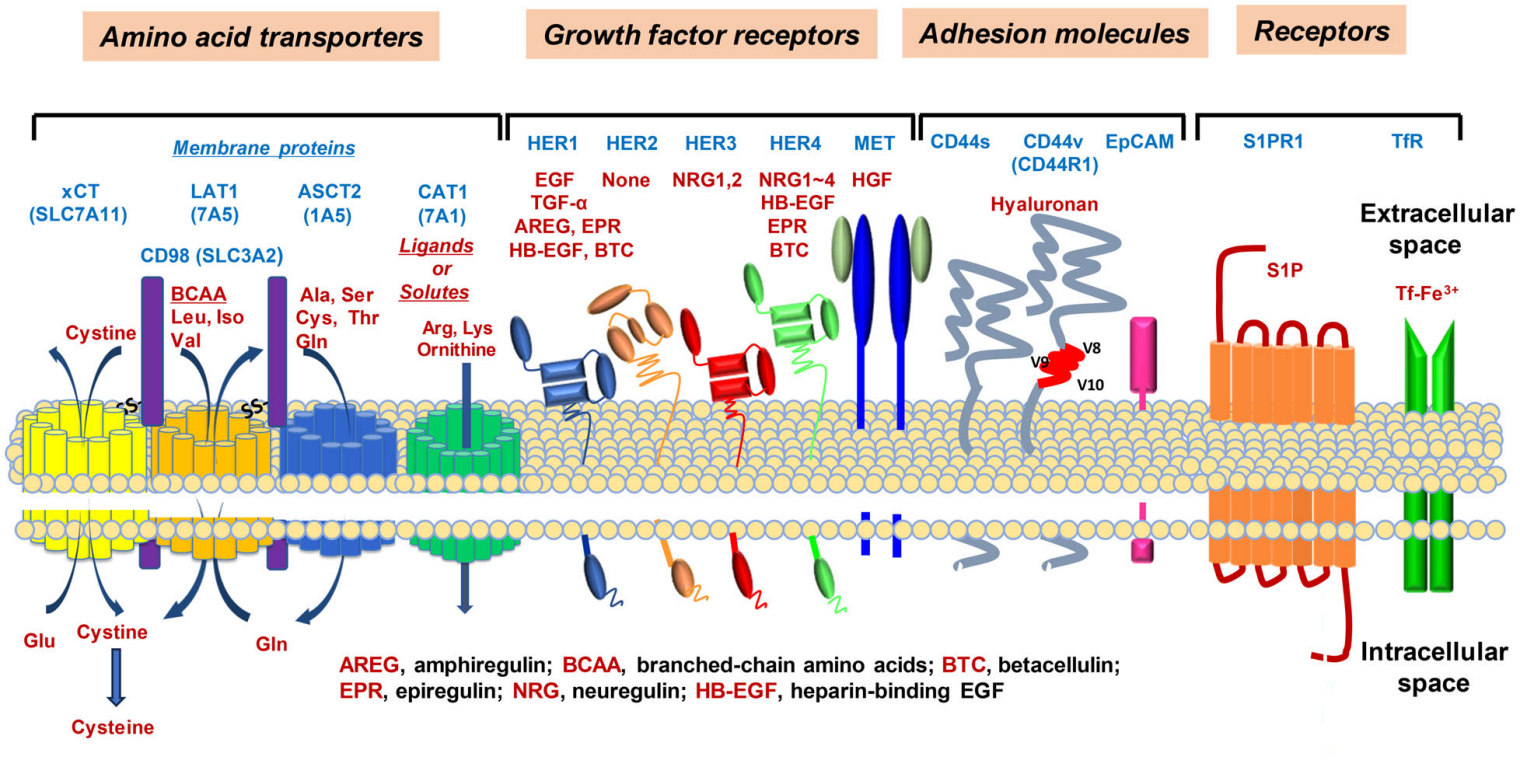
Target molecules for the diagnosis and treatment of PDAC. These membrane proteins (amino acid transporters, growth factor receptors, adhesion molecules, and S1P and Tf receptors) can be targeted by mAbs or low‐molecular‐weight chemicals.

In this comprehensive PDAC study, we characterized *in vitro* and *in vivo* PDAC by specific mAbs against cancer‐associated membrane proteins containing growth factor receptors, transporters and adhesion molecules (Fig. 11). The expression of membrane proteins in pancreatic cell lines was evaluated by FCM. Growth factor receptors (HER1, HER2, HER3, HER4 and MET), amino acid transporters (LAT1, xCT, ASCT2 and CAT1), adhesion molecules (CD44v and EpCAM), TfR and S1PR1 were highly expressed in *in vitro* PDAC cell lines (Fig. 1), but were expressed at low levels in human PBMC (Fig. 2). In comparison with the strong reactivity to human PBMC by mAbs against pan‐CD44, the other mAbs exhibited higher reactivity to PDAC than to PBMC. Regarding CD44, we reported the significance of CD44R1 (CD44v8‐v9‐v10), interacting with an xCT cystine transporter and authorizing cancer stem cells to defense reactive oxygen species by increasing the intracellular glutathione [31], and demonstrated the anti‐tumor effects of fully human mAbs against CD44v8 expressed on cancer stem cells [41]. The effects of S1P‐S1PR1 signaling on lymphocyte trafficking and tissue distribution are well known [56], and this signaling is involved in cancer cell functions [57]. Ovarian cancer cell senescence regulated by S1PR1 has recently been reported [58]. In the present study, we revealed the frequent and high expression of S1PR1 in *in vitro* and *in vivo* PDAC (Figs 1, 6, and 7).

We previously reported the anti‐tumor effects of mAbs against CD44v8 [41], HER3 [19], LAT1 [28], ASCT2 [35] and CAT1 [37] in xenograft models of human head and neck [41], colon [19, 35], uterus cervix [41] and breast [19] human tumors. As a result of unexpectedly high expression of S1PR1 in PDAC (Figs 1 and 7) and its low expression in human PBMC (Fig. 2), we selected anti‐S1PR1 rat mAb [38] in addition to rat mAbs against HER3, ASCT2, and CD44v9 in the analysis of *in vivo* tumor growth inhibition. All four rat mAbs (γ2a, κ) used in the present study markedly inhibited xenografted MIA PaCa‐2 tumor growth (Fig. 5), with anti‐S1PR1 mAb exerting the strongest anti‐tumor effects.

The internalization activity of mAbs against target molecules has been identified as a crucial candidate for anti‐cancer mechanism of mAb therapy [19, 28, 35, 37]. The internalization of various membrane proteins by mAbs, which can lead to the disfunction of target proteins, was observed in the majority of PDAC cell lines (Fig. 3). The reliability of FCM‐based internalization experiments used in the present study has been demonstrated by the coincident data using image analyses with GFP‐fused target proteins [28, 37]. We assume the possibility of mAb‐mediated internalization activity, for the explanation of *in vivo* anti‐PDAC tumor mechanisms. Anti‐HER3, CD44v9 or ASCT2 mAb‐mediated internalization was observed in MIA PaCa 2 (Fig. 3), and we have already reported the internalization activity of anti‐S1PR1 mAb [38]. Antibody‐dependent cellular cytotoxicity (ADCC) with nude mouse splenocytes or human PBMC was also demonstrated by our rat (γ2a) and humanized (γ1) mAb, which are ADCC‐inducible IgG isotypes [28, 37, 41]; therefore, internalization and/or ADCC‐based independent mAb therapy may be applicable and effective for PDAC patients.

To overcome the possible limitations of independent mAb therapy, antibody‐drug conjugates have recently been developed and approved for medical purposes [59]. Regarding the application of antibody‐drug conjugates, we examined the growth inhibitory effects of rat mAbs on PDAC cell lines with toxin‐conjugated secondary antibodies. The inhibitory activity of our various rat mAbs with Saporin‐conjugated anti‐rat IgG against many PDAC cell lines was demonstrated (Fig. 4).

The reactivity of mAbs with in *in vivo* PDAC was evaluated by IHC using pancreatic tissue arrays. Growth factor receptors (HER1, HER2, HER3 and MET), amino acid transporters (LAT1, xCT, ASCT2 and CAT1), CD44v8 and S1PR1 were detected at high frequencies (64–100%) in PDAC tissues using novel rat mAbs. In the IHC score‐based heatmap analysis, membrane proteins aligned into CD44v8, growth factor receptors and transporters, and pancreatic cancers were clearly grouped as two clusters, mainly by CD44v8 expression status. Furthermore, we identified CD44v‐high (stage 3‐rich PDAC) and CD44v‐low (stage 4‐rich NEC) populations by PCA analyses. CD44v‐high PDAC may contain a pancreatic cancer stem cell population [60]. In addition, a positive correlation in the dual expression of proteins, such as HER1‐MET, LAT1‐MET, CD44v8‐MET, LAT1‐ASCT2, xCT‐ASCT2, CAT1‐HER3 and S1PR1‐CD44v8, was suggested. These correlations are novel results for PDAC, whereas those of HER1‐MET [61] and LAT1‐ASCT2 [62] have been reported in various cancers and have contributed to a more detailed understanding of cancer biology and its application to cancer therapy.

In TCGA analyses, the higher mRNA expression of *EGFR*, *ERBB2*, *ERBB3*, *MET* and various amino acid transporters was observed in *CD44v*‐high PDAC than in *CD44v8*‐low PDAC, and the higher mRNA expression of *CD44v8*, *EGFR*, *ERBB2*, *ERBB3*, *MET* and *CD44* correlated with the poor prognosis of PDAC patients. Furthermore, the poor prognosis of patients with *CD44v8/MET* double‐high PDAC (Fig. 10) may fit these combinations of double‐high expression (high IHC scores) on PDAC tissues with higher TNM stages (Fig. 8D).

HER3 has been associated with drug resistance to anti‐HER1 and anti‐HER2 therapies [20, 63], and we previously reported that anti‐HER3 mAb reinforced anti‐HER1 and HER2 therapies [20]. Furthermore, we demonstrated a significant positive correlation between HER3 and MET expression in human colon cancers and indicated the potential of the dual targeting of HER3/MET as a promising cancer therapy [24]. In addition to the association of HER2 with CD98 and bispecific anti‐HER2/CD98 mAb‐mediated growth inhibition of human breast cancers [64], we have recently showed that pan‐HER inhibitions including HER1 and HER4 blockade are more effective than selective targeting of HER2‐HER3 in NRG1 fusion‐positive cancers [65].

Therefore, multi‐targeting cancer therapy may be more effective than single targeting therapy if cancer cells have various target molecules such as growth factor receptors, transporters and adhesion molecules. We propose that CD44v [31, 44], S1PR1 [14, 57], HER3 [19, 20], MET [14, 24] and CD98 [63, 66], LAT1 [27, 30], xCT [31, 33], ASCT2 [14, 35] cancer‐associated amino acid transporters represent novel and promising targets for the diagnoses and treatments of PDAC. Regarding these membrane proteins, therapies with low‐molecular‐weight drugs such as xCT [67] and LAT1 [68] inhibitors will be available in addition to mAb‐based diagnoses and treatments.

## Conclusions

Various types of PDAC (epithelial, mesenchymal and stem cell types) will be handled by targeting mAb‐defined single or combinations of multiple molecules (CD44v, S1PR1, HER3, MET, LAT1, xCT, ASCT2 and CAT1) introduced in the present study.

## Conflicts of interest

The authors declare that they have no conflicts of interest.

### Peer review

The peer review history for this article is available at https://www.webofscience.com/api/gateway/wos/peer‐review/10.1002/2211‐5463.13963.

## Author contributions

TN, KO, SO, SY, SM, HY, KK, YT, KI, YH, KM, KS‐T, NN and TA contributed to the acquisition and analysis of experimental data. TN, KO, SO, TI and TM contributed to the drafting of the manuscript. HS, TI, and TM contributed to the conception and design of this study. All authors read and approved the final version of the manuscript submitted for publication.

## Data Availability

The data that support the findings of this study are available from the corresponding author upon reasonable request.
